# Design of a Photonic Crystal Fiber Optic Magnetic Field Sensor Based on Surface Plasmon Resonance

**DOI:** 10.3390/s25133931

**Published:** 2025-06-24

**Authors:** Yuxuan Yi, Hua Yang, Tangyou Sun, Zao Yi, Zigang Zhou, Chao Liu, Yougen Yi

**Affiliations:** 1 School of Mathematics and Science, Joint Laboratory for Extreme Conditions Matter Properties, The State Key Laboratory of Environment-Friendly Energy Materials, Tianfu Institute of Research and Innovation, Southwest University of Science and Technology, Mianyang 621010, China; 18075122780@163.com; 2 School of Science, Lanzhou University of Technology, Lanzhou 730050, China; hyang@lut.edu.cn; 3 Guangxi Key Laboratory of Precision Navigation Technology and Application, Guilin University of Electronic Technology, Guilin 541004, China; suntangyou@guet.edu.cn; 4 School of Chemistry and Chemical Engineering, Jishou University, Jishou 416000, China; 5 School of Physics and Electronic Engineering, Northeast Petroleum University, Daqing 163318, China; msm-liu@126.com; 6 College of Physics, Central South University, Changsha 410083, China; yougenyi@csu.edu.cn

**Keywords:** fiber optic magnetic field sensor, photonic crystal fiber, magnetofluid or ferrofluid, surface plasmon resonance, sensitivity optimization

## Abstract

To enhance the sensing performance of fiber-optic magnetic field sensors, we explored the design, optimization, and application prospects of a D-type fiber-optic magnetic field sensor. This D-type PCF-SPR sensor is metal coated on one side (the metal used in this study is gold), which serves as the active metal for SPR and enhances structural stability. Magnetic fluid is applied on the outer side of the gold film for SPR magnetic field sensing. Six internal air holes arranged in a hexagonal shape form a central light transmission channel that facilitates the connection between the two modes, which are the sensor’s core mode and SPP mode, respectively. The outer six large air holes and two small air holes are arranged in a circular pattern to form the cladding, which allows for better energy transmission and reduces energy loss in the fiber. In this paper, the finite element method is employed to analyze the transmission performance of the sensor, focusing on the transmission mode. Guidelines for optimizing the PCF-SPR sensor are derived from analyzing the fiber optic sensor’s dispersion curve, the impact of surface plasmon excitation mode, and the core mode energy on sensing performance. After analyzing and optimizing the transmission mode and structural parameters, the optimized sensor achieves a magnetic field sensitivity of 18,500 pm/mT and a resolution of 54 nT. This performance is several orders of magnitude higher than most other sensors in terms of sensitivity and resolution. The SPR-PCF magnetic field sensor offers highly sensitive and accurate magnetic field measurements and shows promising applications in medical and industrial fields.

## 1. Introduction

Earth’s extensive magnetic field makes magnetic field detection technology essential, as all objects on Earth experience varying degrees of magnetization. Recently, rapid advancements in magnetic field sensors have highlighted the growing importance of magnetic fields in various aspects of human life. For instance, in geological exploration, magnetic exploration technology is invaluable for locating underground mineral resources. Many minerals, such as iron ore, are naturally magnetic, allowing geologists to identify rock types and geological structures and predict potential mining areas [1]. In medical diagnosis, detecting weak magnetic field signals from the brain, heart, and other body parts can aid in diagnosing conditions such as coronary heart disease and cerebral thrombosis [2,3]. In marine applications, magnetic field sensors can be used to explore seabed mineral resources and monitor ocean magnetic fields for tsunami warning systems [4]. Additionally, these sensors contribute to marine biological research [5], underwater archaeology [6], shipwreck detection, and other applications [7,8]. Thus, advancing magnetic field sensor technology is highly significant.

Currently, the primary magnetic field sensors include electrical types such as magnetoresistive sensors [9], atomic magnetometers [10], fluxgate sensors [11], and detection coils [12]. Although these conventional electrical sensors have diverse applications, they suffer from disadvantages including large size, high cost, high power consumption, and poor resistance to electromagnetic interference. Additionally, these sensors are prone to electromagnetic interference, leading to increased noise in the magnetic field signal. These limitations restrict electrical sensors to harsh conditions, such as superconducting quantum interferometers needing liquid nitrogen cooling [13] and spin-exchange relaxation-free atomic magnetometers functioning only near zero magnetic fields [14,15].

As science and technology advance, the demand for magnetic field sensors is growing, with modern requirements emphasizing high sensitivity, compact size, fast response, low cost, and strong anti-jamming capabilities. Therefore, there is a need to develop advanced magnetic field sensors. Among various sensor types, fiber optic sensors offer significant advantages over traditional sensors. This technology leverages the light propagation properties of optical fibers to measure a range of physical quantities, including temperature and strain. The fundamental principle involves monitoring changes in the intensity, wavelength, or phase of light as it travels through the optical fiber, enabling accurate and sensitive measurements [16]. Therefore, optical sensors utilizing fiber optic sensing technology present numerous advantages, including compact size, robust immunity to electromagnetic interference, capabilities for remote sensing, higher sensitivity, and ease of integration and multiplexing [17]. Because of these advantages, the fiber optic sensor not only can be larger, with excessive power consumption and weak resistance to electromagnetic interference and other shortcomings of the traditional electrical sensors, but also its application environment is more extensive and can be applied in complex and harsh environments. This paper presents an optical fiber magnetic field sensor based on surface plasmon resonance (SPR) [18]. SPR is an electromagnetic mode formed by the interaction of free electrons and photons in the interface region between metal and dielectric [19,20,21]. When a magnetic field is applied to this metal film, it alters the film’s optical properties, thus affecting the SPR effect [22,23,24].

Photonic Crystal Fiber (PCF) is an innovative optical fiber model that transmits light by incorporating periodically arranged air holes within the fiber [25]. The arrangement of air holes within a PCF can result in varying optical properties [26]. Metal film surface plasmon resonance (SPR) coatings leverage PCF properties and offer significantly improved sensing performance compared to traditional SPR sensors [27]. Recently, SPR-PCF sensors have been extensively used to measure various parameters, including refractive index and magnetic fields [28,29,30].

In this paper, SPR and PCF are combined, and the optical fiber structure is designed as a D-type structure so as to obtain a high-sensitivity optical fiber magnetic field sensor. A side circular paraboloid was selected to create an open-loop channel on one side of the sensor. Select a metal film (gold film) on one side of the sensor, and magnetic fluid surrounds the optical fiber for SPR-based magnetic field sensing, achieving high sensitivity. The sensor is evaluated through the finite element method (FEM) analysis, with investigations into its transmission mode and structural parameters. The results demonstrate that the fiber optic sensor exhibits high magnetic field sensitivity and is well-suited for applications in navigation, earthquake warning, and industrial production. Additionally, it is effective for detecting weak magnetic field changes.

## 2. Model and Principle

**Figure 1:**
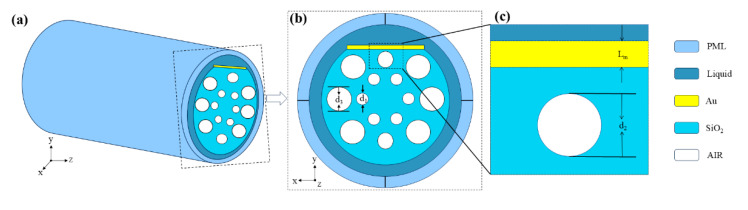
(**a**) A 3D schematic of a Model D PCF-SPR Magnetic Field Sensor; (**b**) a cross-sectional view of the D-type sensor; (**c**) detail of the structure of the surface of the proposed fiber sensor.

Figure 1 shows the model diagram of the D-type fiber-optic magnetic field sensor developed in this study. One side of the sensor is coated with the metallic substance gold, which serves as the active metal for SPR and enhances structural stability [22]. Magnetic fluid is applied outside the gold film for SPR magnetic field sensing. Six inner air holes are arranged hexagonally, with the center forming a light transmission channel to guide coupling between the core mode and SPP mode [31]. The cladding is formed by the placement of the two tiny and six big air holes around a circular pattern, effectively constraining energy leakage from the core. This configuration constitutes a D-type PCF-SPR magnetic field sensor. In Figure 1, d_1_, d_2_, d_3_, and L_m_ denote the diameters of air holes 1, 2, and 3 and the gold film thickness, respectively. Sensor structure parameterization in Table 1. All numerical simulations were performed using COMSOL Multiphysics 6.1, employing the Wave Optics Module based on the finite element method (FEM) [32,33,34]. The 2D cross-sectional structure of the PCF sensor was constructed, and the electromagnetic wave propagation was simulated under frequency domain conditions. Material properties, including the complex refractive index of gold, were assigned using empirical data [35]. A fine mesh was applied near the metal–dielectric interface to ensure accurate resolution of surface plasmon fields. Perfectly matched layers (PMLs) were used to absorb outgoing waves and avoid boundary reflections [36,37,38].

COMSOL Multiphysics was used in this study, and we set the refractive index of the air at the internal no-material to 1. The refractive index of the magneto-fluid was defined using the Langevin function, and silicon dioxide was chosen as the optical fiber’s inner material, with its refractive index determined by the Sellmeier formula [39].

(1) $$n^{2}\left(\lambda \right)=1+\frac{B_{1}\lambda^{2}}{\lambda^{2}-C_{1}}+\frac{B_{2}\lambda^{2}}{\lambda^{2}-C_{2}}+\frac{B_{3}\lambda^{2}}{\lambda^{2}-C_{3}}$$

where *λ* is the set operating wavelength, and the constants *B*_1_ = 0.696166, *B*_2_ = 0.407942, *B*_3_ = 0.897479, *C*_1_ = 0.068404 μm, *C*_2_ = 0.116241 μm, and *C*_3_ = 9.896161 μm in the Sellmeier equation.

In addition, the dielectric function of the plated gold film was defined through the Drude–Lorentz model [40]:

(2) $$\varepsilon \left(\omega \right)=\varepsilon_{\infty }-\frac{\omega_{D}^{2}}{\omega \left(\omega +j\gamma_{D}\right)}+\frac{\Delta \varepsilon \cdot \Omega_{L}^{2}}{\left(\omega^{2}-\Omega_{L}^{2}\right)+j\Gamma_{L}\omega }$$

where $\omega_{D}$ = 4227.2π THz represents the plasma frequency, $\varepsilon_{\infty }$ = 5.9673 is the permittivity at a high frequency, $\gamma_{D}$ = 31.84π THz represents the damping frequency, $\Omega_{L}$ = 1300.14π THz represents the oscillator strength, $\Gamma_{L}$ = 209.72π THz corresponds to the spectral width of the Lorentzian oscillator, and ∆*ε* = 1.09 is the weighting factor of the Lorentzian oscillator.

In the present study, perfectly matched layer and scattering boundary conditions were implemented. When the core mode and the surface plasmon-polarized exciton (SPP) mode are phase-matched, core-guided light couples to the surface plasmon on the gold film [22,41]. In this study, loss spectrum analysis is used to characterize the designed fiber optic magnetic field sensor. In the SPR-PCF sensor, energy coupling occurs when the two modes, the core of the fiber and the SPP on the metal surface, are phase-matched under certain conditions, transferring energy from the core to the SPP [42]. The fiber core mode loses energy as a result of this energy transfer, producing SPR peaks at specific wavelengths, which are observed in the loss spectrum. Limited light confinement by the PCF cladding causes some light to escape from the core region to the cladding, leading to loss. This loss primarily results from inadequate light confinement by the PCF cladding, causing light to propagate from the core into the cladding. It is possible to assess the developed sensor’s performance by looking at the loss spectrum. Changes in the loss spectrum may be used to infer information by computing the loss value from the imaginary component of the fiber core’s effective refractive index, hence allowing the sensing function.

The refractive index expression resulting from the analysis of the designed model by the simulation software COMSOL Multiphysics 6.1 is given by [43]:

(3) neff=Reneff+Imneff

where Re(*n_eff_*) is the real part of the effective refractive index and Im(*n_eff_*) is the imaginary part of the effective refractive index.

The magnitude of the loss value can be calculated from the imaginary part of the effective refractive index Im(*n_eff_*) of the fiber core pattern [44]:

(4) αloss=8.686×2πλIm(neff)×104(dB/cm)

where *λ* is the incident wavelength.

The SPR-PCF sensor is highly sensitive to changes in refractive index since the phase matching between the two analyzed primary modes in the designed sensor is strongly influenced by the refractive index. Wavelength sensitivity quantifies this by expressing it as the ratio of the resonant wavelength shift to the change in the refractive index of the measured object [45,46]. When the sensor’s external magnetic field changes and thus affects the object under test, its corresponding refractive index changes, which alters the surface plasmon resonance wave vector and shifts the center wavelength of the SPR peak in the spectrum [47,48]. By tracking this shift, the corresponding wavelength sensitivity of the sensor can be calculated. The formula is as follows [49]:

(5) $$S_{H}=\frac{\Delta \lambda_{peak}}{\Delta H}(nm/Oe)$$

where ∆*H* is the degree of variation in the external magnetic field reinforcement and ∆*λpeak* is the wavelength drift distance of the SPR peak center.

The change of the refractive index of MF can be expressed by the Langevin model function as [50]:

(6) $$n_{mf}=(n_{s}-n_{0})[\coth(\alpha \frac{H-H_{c,n}}{T})-\frac{T}{\alpha (H-H_{c,n})}]+n_{0}$$

where *n_s_* is the saturation value of the refractive index, *n*_0_ is the initial refractive index of MF, *α* is the fitting coefficient, *T* is the temperature, *H_c,n_* is the threshold range of the magnetic field, and *H* is the external magnetic field. Different concentrations, temperatures, and particle sizes of MF will affect the refractive index of MF. The initial parameters are set to *T* = 297.45 K, *α* = 5, *H_c,n_* = 30 Oe, *n*_0_ = 1.4352, and *n_s_* = 1.4385.

## 3. Simulations and Analysis

**Figure 2:**
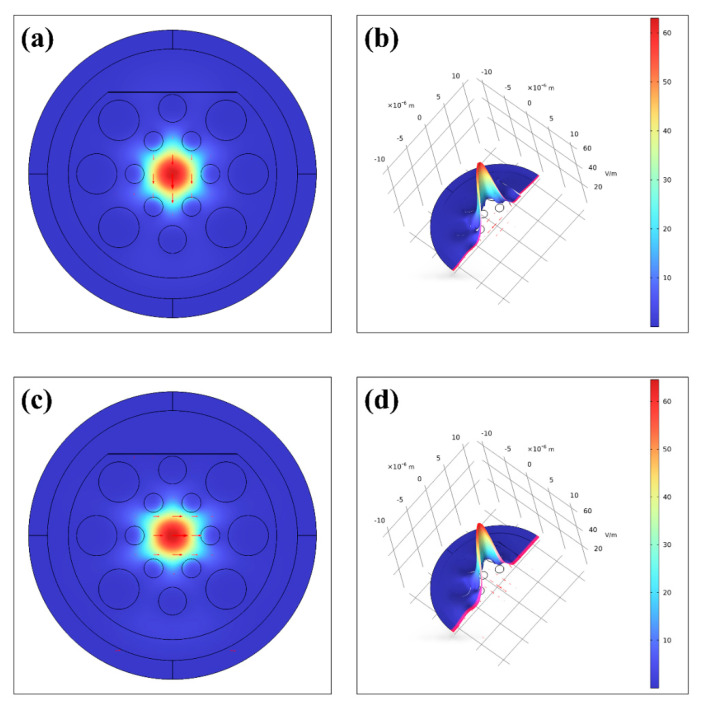
(**a**) Y−polarized core mode; (**b**) Y−polarized electric field distribution; (**c**) X−polarized core mode; and (**d**) X−polarized electric field distribution.

Since the structure of the PCF designed in this study is asymmetric in cross-section, it leads to different propagation constants and field distributions for modes with different polarization directions, which results in a different distribution of refractive indices of the sensors in x and y directions, which further leads to the generation of x-polarized fiber core modes and y-polarized fiber core modes with different effective refractive indices. Therefore, in this paper, the MF with refractive index *n* = 1.40 and wavelength *wl* = 2.4 μm is taken for simulation, and the core patterns in the x direction and y direction are preliminarily analyzed during the simulation, as shown in Figure 2.

Since the structures designed in this study do not have central symmetry, the two models are quite different. Figure 2 shows that the core is not orthogonal to the coated metal in the x-direction and therefore has minimal interaction with the active metal and is less susceptible to SPR. In contrast, the core in the y-direction is more orthogonal to the gold film and therefore produces a more pronounced SPR phenomenon [51,52]. Therefore, the y-polarized core mode is chosen as the main transmission mode for the sensor designed in this paper.

**Figure 3:**
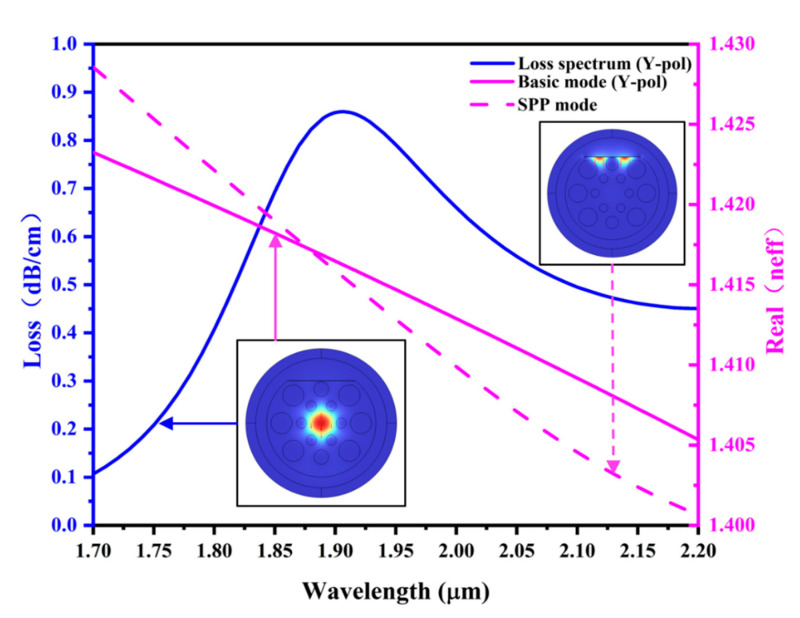
Dispersion curves for the SPP mode and the y−polarized core mode when SPR occurs for the sensor designed in this paper and the loss spectrum in the y−polarized core mode.

As shown in Figure 3, this study explores the connection between the two modes and the refractive index of the MF in order to further investigate the core mode in the y-direction. It does this by analyzing the loss spectrum of the sensor.

Figure 3 shows that the SPR phenomenon occurs when the real part of the refractive index corresponding to the y-core mode matches the real part of the refractive index corresponding to the SPP mode, resulting in a sharp loss peak. Calculations reveal that the intersection of the two mode curves occurs near the SPR peak [53]. This transmission characteristic suggests that the effective refractive index of the two modes described above will vary with the refractive index of the magnetic fluid in the material. Consequently, this will shift the SPR peak so that the sensitivity can be calculated using the appropriate formula.

## 4. Analysis of Nanostructure Parameters

### 4.1. Effect of Structural Parameter Variations on Sensing Characteristics

The transmission characteristics show that, under phase-matching conditions, the effective refractive index of the SPP mode and the y-polarized fiber core mode predominantly determine mode coupling. This index is influenced not only by the materials used but also by the sensor’s structural parameters. Therefore, this study examines the effects of three structural parameters—gold film thickness (L_m_), inner aperture size (d_1_), and outer aperture size (d_3_)—on sensor performance.

#### 4.1.1. Gold Film Thickness

This subsection analyzes how the thickness (L_m_) of the gold film affects sensor performance. Since the metal film is an important condition for exciting the SPR effect, when no metal layer is added, the loss spectrum will become almost flat, the sharp SPR formant peaks will disappear, that is, the surface plasmon exciton will no longer occur, and the total loss value will be significantly reduced [54]. Therefore, during the simulation process, gold films with thicknesses of 50 nm, 55 nm, 60 nm, and 65 nm were tested, with the refractive index fixed at *n* = 1.40.

**Figure 4:**
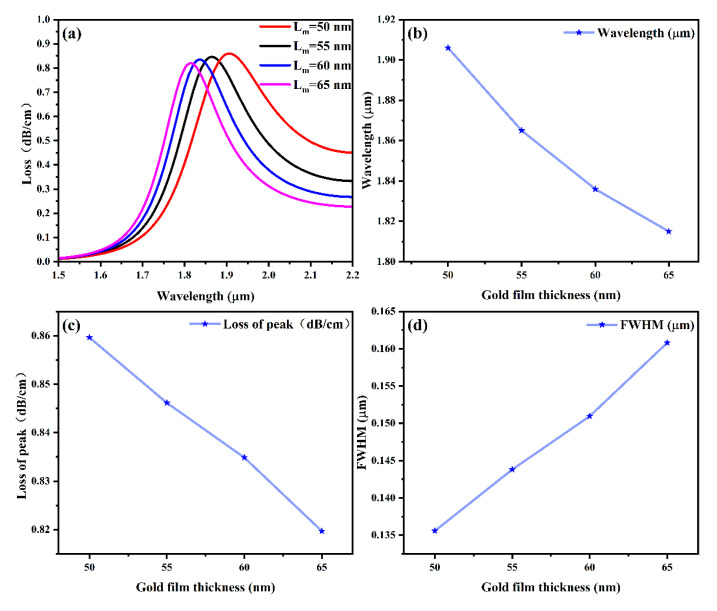
(**a**) Effect of gold film thickness L_m_ on the loss spectrum; (**b**) wavelength of SPR peak corresponding to gold film thickness; (**c**) loss value of SPR peak corresponding to gold film thickness; and (**d**) the thickness of the gold film corresponding to the FWHM of the sensor.

Figure 4a shows that, at all refractive indices of *n* = 1.40, we can clearly find the effect of the coated gold film on the loss spectrum. As the thickness gradually goes from 50 nm to 65 nm, the center wavelength corresponding to the SRP peak is constantly shifted in a shorter direction (blueshift). It can be specifically demonstrated through Figure 4b–d. In Figure 4b, as the thickness of the gold film increases from 50 nm to 65 nm, the central wavelength corresponding to the wave peak shows a decreasing trend, while in the corresponding Figure 4d, the specific FWHM shows an increasing trend; the FWHM becomes wider. Meanwhile, Figure 4c indicates that the loss value corresponding to its peak is also showing a downward trend. These trends indicate that thinner gold films are more suitable for the designed sensors [55,56]. This is due to the SPR fiber optic sensor’s characteristics, where the SPP mode’s dispersion curve exhibits nonlinear changes, enhancing sensitivity and improving overall sensing performance [57,58]. Therefore, a gold film thickness of 50 nm was selected for the sensor.

#### 4.1.2. Inner Aperture Size

**Figure 5:**
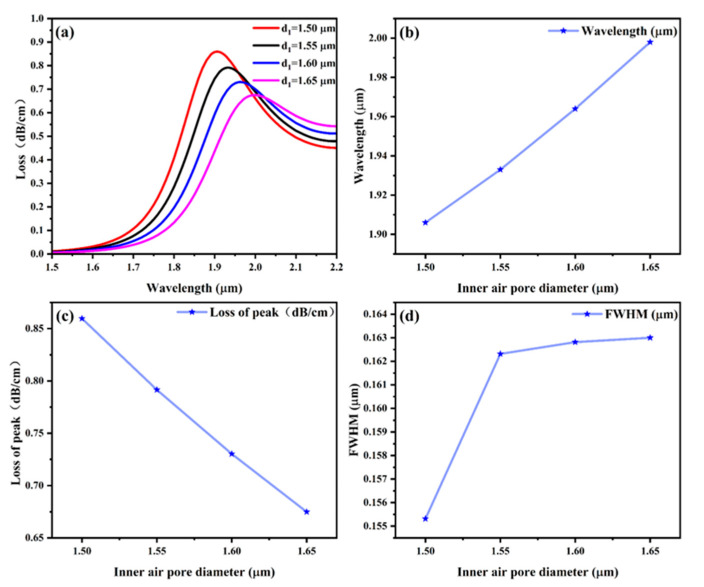
(**a**) Effect of inner air aperture diameter d_1_ on the loss spectrum; (**b**) the wavelength of the SPR peak corresponding to the inner air aperture diameter d_1_; (**c**) loss value of the SPR peak corresponding to the inner air aperture diameter d_1_; and (**d**) the inner air aperture diameter d_1_ corresponding to the FWHM of the sensor.

The effect of the innermost air aperture diameter (d_1_) on the sensor performance is investigated in this part. Firstly, we set the thickness of the gold to 50 nm, and the y-core mode’s effective refractive index was set at *n* = 1.40, and air apertures with dimensions of 1.50 μm, 1.55 μm, 1.60 μm, and 1.65 μm were employed throughout the simulation. Figure 5a illustrates that the SPR peak wavelength in the y-core mode shifts to longer wavelengths (red-shifts) as the inner air aperture diameter (d_1_) increases while keeping the effective refractive index constant [59,60]. Figure 5b indicates that as the radius increases from 1.5 μm to 1.65 μm, the central wavelength corresponding to the wave crest shows an upward trend. However, in Figure 5c, the loss values corresponding to the wave peaks show a downward trend. At the same time, it can be seen that in Figure 5d, FWHM shows a slightly widening trend. These trends indicate that appropriately increasing the radius is more suitable for the designed sensor. The larger the inner aperture, the narrower the light transmission channel, which narrows the light transmission channel and enhances the coupling inside the sensor, thus increasing the sensitivity [61,62]. Therefore, this study selects an inner air hole diameter of 1.65 μm.

#### 4.1.3. Outer Aperture Size

**Figure 6:**
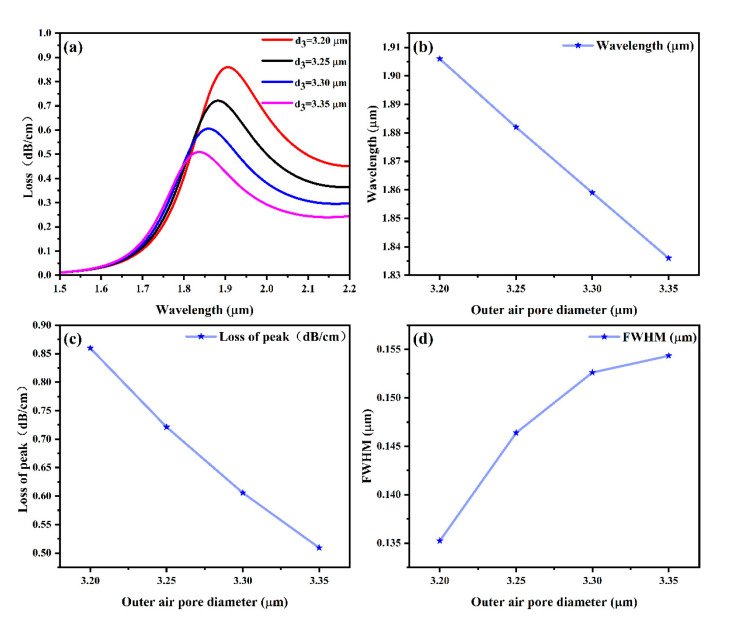
(**a**) Effect of outer air aperture diameter d_1_ on the loss spectrum; (**b**) the wavelength of the SPR peak corresponding to the outer air aperture diameter d_1_; (**c**) the loss value of the SPR peak corresponding to the outer air aperture diameter d_1_; and (**d**) the outer air aperture diameter d_1_ corresponding to the FWHM of the sensor.

Finally, this paragraph concludes by analyzing how sensor performance is affected by the outermost air hole diameter (d_3_). In the tests, air holes with dimensions of 3.20 μm, 3.25 μm, 3.30 μm, and 3.35 μm were conducted, and the y-polarized fiber core mode’s effective refractive index was set at *n* = 1.40, with a gold thickness of 50 nm. The variation in loss values with wavelength is plotted in Figure 6.

Figure 6 shows (a) that the peak SPR wavelengths of the fiber core modes in the y-direction change to shorter wavelengths (blue shift) with increasing diameter of the sensor’s outer aperture (d_3_) with a fixed effective refractive index. In Figure 6b, as the radius increases from 3.2 μm to 3.35 μm, the central wavelength corresponding to the wave crest shows a decreasing trend. In the corresponding Figure 6d, the specific FWHM shows an increasing trend; the FWHM becomes wider. Meanwhile, it can be seen from Figure 6c that the loss value corresponding to its peak also shows a downward trend. These trends indicate that a smaller outer pore radius is more suitable for the designed sensor. This reduces the interaction between these modes and weakens the coupling effect, ultimately decreasing sensor sensitivity [63,64]. Therefore, this study selects an outer air hole diameter of 3.20 μm.

### 4.2. Sensing Characterization of Sensors

**Figure 7:**
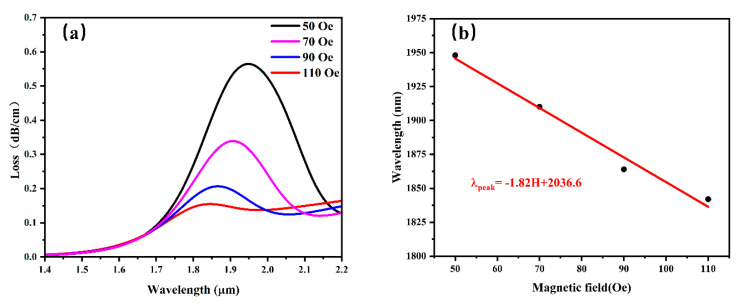
(**a**) Loss spectra of the optimized fiber optic sensor at different magnetic fields, 50–110 Oe; (**b**) linear fitting results of SPR center wavelengths for the y−core mode at various magnetic fields.

The D-type SPR-PCF sensor’s magnetic field characterization was carried out after the sensor’s parameters were optimized. Magnetic field strengths of 50 Oe, 70 Oe, 90 Oe, and 110 Oe were used for the tests. The results are displayed in Figure 7. Figure 7 illustrates how the magnetic fluid’s refractive index increases as the strength of the external magnetic field increases, raising the SPP mode’s refractive index and resulting in a blueshift in the SPR peak wavelength [65,66,67]. Equation (5) is used to compute the sensor’s sensitivity, which is displayed in Figure 7 and comes out to 18,200 pm/mT. Although Equation (5) yields sensitivity in units of nm/Oe, we convert and report the values in pm/mT, which are more commonly used in magnetic field sensing applications. The conversion factor is 1 nm/Oe = 10,000 pm/mT, considering that 1 Oe ≈ 0.1 mT. This unit conversion does not affect the validity of the results and facilitates practical interpretation. In addition, we also calculated the resolution of the sensor, which is 54 nT. This suggests that the sensor is capable of detecting nanotesla-scale changes in the magnetic field.

In order to compare the sensitivity effect of this sensor, this paper also summarizes the sensitivity of the following magneto-fluid-based fiber optic sensors, as shown in Table 2 [68,69,70,71,72,73]. The magnetic field sensitivity and the resolution of the magnetic field response of the D-type SPR-PCF sensor designed in this paper are several orders of magnitude higher than those of most other types of sensors, as can be seen from the comparison of the sensor sensitivity in the table. Thanks to the higher sensitivity, the sensor developed in this work is not limited to use in navigation, earthquake early warning, and industrial production but also in the fields that need to respond to weak magnetic field changes.

## 5. Conclusions

This study outlines the working principle of the sensor design after providing an overview of the basic concepts of surface plasmon resonance. The sensing characteristics are discussed next, using loss spectrum analysis, along with the methodological formulas for assessing the performance of the sensor. Finally, the study introduces the magnetic fluid, the substance under measurement, and describes its optical properties. A new D-type SPR-PCF sensor is proposed. This sensor is metal-coated on one side (gold), which serves as the active metal for SPR and enhances structural stability. Magnetic fluid is applied outside the gold film for SPR magnetic field sensing. The hexagonal arrangement of the six innermost air holes creates a light transmission channel within the hexagon. The outer six large air holes and two smaller ones are arranged in a circular pattern to form the PCF cladding. Additionally, perfectly matched layers and scattering boundary conditions were implemented. The sensor’s gold film thickness and air hole parameters were optimized and analyzed to achieve the best sensing performance. The optimized sensor achieves a magnetic field sensitivity of 18,500 pm/mT and a resolution of 54 nT, significantly outperforming most other sensors in both sensitivity and resolution.

## Figures and Tables

**Table 1:** Sensor structure parameterization.

Notation	Designation	Parameter Value
d_1_	Aperture 1	1.5 μm
d_2_	Aperture 2	2.2 μm
d_3_	Aperture 3	3.2 μm
L_m_	Gold Film Thickness	50–65 nm

**Table 2:** Comparison of the sensitivity of magnetic field sensors with fiber optic structures from the other literature.

Notation	Designation	Sensitive
Model A [68]	LPFG [68]	176.4 pm/mT
Model B [69]	PM-PCF [69]	242 pm/mT
Model C [70]	PCF-FP [70]	330 pm/mT
Model D [71]	taper fiber-SPR [71]	4400 pm/mT
Model E [72]	PS-FBG [72]	24.2 pm/mT
Model F [73]	PCF-SPR [73]	612.5 pm/mT
Model of this work	D-SPR-PCF	67.45 pm/mT

## Data Availability

Publicly available datasets were analyzed in this study. This data can be found here: [https://www.lumerical.com/] (accessed on 1 January 2020).

## References

[1] Tinto K.J., Padman L., Siddoway C.S., Springer S.R., Fricker H.A., Das I., Tontini F.C., Porter D.F., Frearson N.P., Howard S.L. (2019). Ross Ice Shelf response to climate driven by the tectonic imprint on seafloor bathymetry. Nat. Geosci..

[2] Liu H., Hu D.J.J., Sun Q., Wei L., Li K., Liao C., Li B., Zhao C., Dong X., Tang Y. (2023). Specialty optical fibers for advanced sensing applications. Opto-Electron. Sci..

[3] Wu G.X., Zhu R.Z., Lu Y.Q., Hong M., Xu F. (2024). Optical scanning endoscope via a single multimode optical fiber. Opto-Electron. Sci..

[4] Satake K., Atwater B.F. (2007). Long-Term Perspectives on Giant Earthquakes and Tsunamis at Subduction Zones. Annu. Rev. Earth Planet. Sci..

[5] Imas J.J., Matías I.R., Del Villar I., Ozcáriz A., Zamarreño C.R., Albert J. (2023). All-fiber ellipsometer for nanoscale dielectric coatings. Opto-Electron. Adv..

[6] Guerneve T., Subr K., Petillot Y. (2018). Three-dimensional reconstruction of underwater objects using wide-aperture imaging SONAR. J. Field Robot..

[7] Liu S.H., Yang H., Tang C.J., Yi Z., Yi Y.G., Wang J.Q., Li B.X. (2025). Highly sensitive photonic crystal optic fiber with annular stomatal arrangement for cervical cancer cell detection. Phys. Lett. A.

[8] Yin S.Y., Guo Q., Liu S.R., He J.W., Yu Y.S., Tian Z.N., Chen Q.D. (2024). Three-dimensional multichannel waveguide grating filters. Opto-Electron. Sci..

[9] Guo L., Zhi S., Sun X., Lei C., Zhou Y. (2017). Ultrasensitive detection of bioanalytes based on signal amplification of coil-integrated giant magnetoimpedance biosystems. Sens. Actuators B Chem..

[10] Zhang L., Zhen Y., Tong L. (2024). Optical Micro/Nanofiber Enabled Tactile Sensors and Soft Actuators: A Review. Opto-Electron. Sci..

[11] Chitarin G., Aprile D., Brombin M., Marconato N., Svensson L. (2017). Feasibility study of a flux-gate magnetic field sensor suitable for ITER Neutral Beam Injectors. Fusion Eng. Des..

[12] Poliakov S.V., Reznikov B.I., Shchennikov A.V., Kopytenko E.A., Samsonov B.V. (2017). The range of induction-coil magnetic field sensors for geophysical explorations. Seism. Instr..

[13] Yan X., Lin Q., Wang L., Liu G.D. (2022). Active absorption modulation by employing strong coupling between magnetic plasmons and borophene surface plasmons in the telecommunication band. J. Appl. Phys..

[14] Kominis I., Kornack T., Allred J., Romalis M.V.J.N. (2003). A subfemtotesla multichannel atomic magnetometer. Nature.

[15] Shah V., Knappe S., Schwindt P.D.D., Kitching J. (2007). Subpicotesla atomic magnetometry with a microfabricated vapour cell. Nat. Photonics.

[16] Culshaw B., Kersey A. (2008). Fiber-optic sensing: A historical perspective. J. Lightw. Technol..

[17] Jiang B., Hou Y., Wu J., Ma Y., Gan X., Zhao J. (2023). In-fiber photoelectric device based on graphene-coated tilted fiber grating. Opto-Electron. Sci..

[18] Rodríguez-Schwendtner E., Díaz-Herrera N., Navarrete M., González-Cano A., Esteban Ó. (2017). Plasmonic sensor based on tapered optical fibers and magnetic fluids for measuring magnetic fields. Sens. Actuators A Phys..

[19] Wang H.Y., Ma R., Liu G.D., Wang L.L., Lin Q. (2023). Optical force conversion and conveyor belt effect with coupled graphene plasmon waveguide modes. Opt. Express.

[20] Ai Z., Liu H.F., Cheng S.B., Zhang H.F., Yi Z., Zeng Q.D., Wu P.H., Zhang J.G., Tang C.J., Hao Z.Q. (2025). Four peak and high angle tilted insensitive surface plasmon resonance graphene absorber based on circular etching square window. J. Phys. D Appl. Phys..

[21] Ma R., Zhang L., Liu G., Wang L., Lin Q. (2021). The total optical force exerted on black phosphorus coated dielectric cylinder pairs enhanced by localized surface plasmon. J. Appl. Phys..

[22] Lameirinhas R.A.M., Torres J.P.N., Baptista A., Martins M.J.M. (2022). A New Method to Analyse the Role of Surface Plasmon Polaritons on Dielectric-Metal Interfaces. IEEE Photonics J..

[23] Lameirinhas R.A.M., Torres J.P.N., Baptista A., Martins M.J.M. (2021). The impact of nanoantennas on ring resonators’ performance. Opt. Commun..

[24] Li W., Cheng S., Zhang H., Yi Z., Tang B., Ma C., Wu P., Zeng Q., Raza R. (2024). Multi-functional metasurface: Ultra-wideband/multi-band absorption switching by adjusting guided mode resonance and local surface plasmon resonance effects. Commun. Theor. Phys..

[25] Cerqueira S.A. (2010). Recent progress and novel applications of photonic crystal fibers. Rep. Prog. Phys..

[26] Gao H., Hu H.F., Zhan Q.W. (2025). Tailoring temperature response for a multimode fiber. Opto-Electron Sci.

[27] Zhang S.W., Yang H., Tang C.J., Yi Z., Zhang J.G., Wang J.Q., Li B.X. (2025). Multiple tunable six-peak graphene absorber for high-performance refractive index sensing. Phys. B Condens. Matter.

[28] Rifat A.A., Ahmed R., Yetisen A.K., Butt H., Sabouri A., Mahdiraji G.A., Yun S.H., Adikan F.M. (2017). Photonic crystal fiber based plasmonic sensors. Sens. Actuators B Chem..

[29] Hu J.Y., Tan C.X., Bai W.D., Li Y.M., Lin Q., Wang L.L. (2022). Dielectric nanocavity-coupled surface lattice resonances for high-efficiency plasmonic sensing. J. Phys. D Appl. Phys..

[30] Rajeswari D., Revathi A.A. (2022). Highly sensitive SPR-based PCF bio sensor for plasma cell detection in human blood for the detection of early stage cancer. Optik.

[31] Li Z.T., Li X., Liu G.D., Wang L.L., Lin Q. (2023). Analytical investigation of unidirectional reflectionless phenomenon near the exceptional points in graphene plasmonic system. Opt. Express.

[32] Zhang B.W., Luo Y.N. (2025). Dynamic optical tuning and sensing in L-shaped dirac semimetal-based terahertz metasurfaces. Phys. Lett. A.

[33] Li W.X., Cheng S.B., Yi Z., Zhang H.F., Song Q.J., Hao Z.Q., Sun T.Y., Wu P.H., Zeng Q.D., Raza R. (2025). Advanced optical reinforcement materials based on three-dimensional four-way weaving structure and metasurface technology. Appl. Phys. Lett..

[34] Yang C., Luo M.H., Ju X.W., Hu J.Y. (2024). Ultra-narrow dual-band perfect absorber based on double-slotted silicon nanodisk arrays. J. Phys. D Appl. Phys..

[35] Chen S., Wu X.H., Fu C.J. (2024). Active tuning of anisotropic phonon polaritons in natural van der Waals crystals with negative permittivity substrates and its application in energy transport. Opto-Electron. Sci..

[36] Shao L.X., Yang H., Yi Z., Wang J.Q., Tang C.J., Deng J., Li B.X. (2025). Graphene Terahertz Metamaterials Absorber with Multiple Absorption Peaks and Adjustable Incident Polarization Angle. Phys. B Condens. Matter.

[37] Ma R., Zhang L.G., Zeng Y., Liu G.D., Wang L.L., Lin Q. (2023). Extreme enhancement of optical force via the acoustic graphene plasmon mode. Opt. Express.

[38] Li Z.T., Cheng S.B., Zhang H.F., Yang W.X., Yi Z., Yi Y.G., Wang J.Q., Ahmad S., Raza R. (2025). Ultrathin broadband terahertz metamaterial based on single-layer nested patterned graphene. Phys. Lett. A.

[39] Luo M.H., Hu J.Y., Li Y.M., Bai W.D., Zhang R.L., Lin Q., Wang L.L. (2023). Anapole-assisted ultra-narrow-band lattice resonance in slotted silicon nanodisk arrays. J. Phys. D Appl. Phys..

[40] Yang Q., Yu M., Chen Z., Ai S., Kentsch U., Zhou S., Jia Y., Chen F., Liu H. (2025). A novel approach towards robust construction of physical colors on lithium niobate crystal. Opto-Electron. Adv..

[41] Liu H.F., Li J.J., Yang H., Wang J.Q., Li B.X., Zhang H., Yi Y.G. (2025). TiN-Only Metasurface Absorber for Solar Energy Harvesting. Photonics.

[42] Liu Y.J., Liu M.S., Yang H., Yi Z., Zhang H., Tang C.J., Deng J., Wang J.Q., Li B.X. (2025). Photoelectric simulation of perovskite solar cells based on two inverted pyramid structures. Phys. Lett. A.

[43] Liu M.L., Li B.X., Zeng L.L., Wei Y., Wen R.Q., Zhang X.J., Deng C.S. (2023). Dynamic tunable narrow-band perfect absorber for fiber -optic communication band based on liquid crystal. J. Phys. D Appl. Phys..

[44] Hu J.Y., Bai W.D., Tan C.X., Li Y.M., Lin Q., Wang L.L. (2022). Highly electric field enhancement induced by anapole modes coupling in the hybrid dielectric-metal nanoantenna. Opt. Commun..

[45] Guo X.C., Tang C.J., Yi Z., Cheng S.B., Wang J.Q., Li B.X. (2025). Design and application of multi-absorption and highly sensitive monolayer graphene microstructure absorption devices located at terahertz frequencies. Curr. Appl. Phys..

[46] Zeng Z.L., Liu H.F., Zhang H.F., Cheng S.B., Yi Y.G., Yi Z., Wang J.Q., Zhang J.G. (2025). Tunable ultra-sensitive four-band terahertz sensors based on Dirac semimetals. Photonics Nanostruct.-Fundam. Appl..

[47] Tan Z.Q., Lin Q., Du W.J., Wang L.L., Liu G.D. (2025). Simultaneously enhance electric and magnetic Purcell factor by strong coupling between toroidal dipole quasi-BIC and electric dipole. J. Appl. Phys..

[48] Wang J.Q., Sun J.Y., Sun S., Zhang H., Wang Q.Q., Yang J.Y., Mei Y.W. (2025). Numerical simulation of electromagnetically induced transparency in composite metamaterial. Phys. Scr..

[49] Zeng L.L., Li B.X., Wen R.Q., Zhang X.J. (2023). Plasmonic Sensor Based on Multi Fano Resonance in Inverse T Shape Structure for Detection of CO2 Concentration. IEEE Photonics J..

[50] Yang S., Chen Y., Horng H., Hong C.-Y., Tse W., Yang H. (2002). Magnetically-modulated refractive index of magnetic fluid films. Appl. Phys. Lett..

[51] Zhang Y.X., Lin Q., Yan X.Q., Wang L.L., Liu G.D. (2024). Flat-band Friedrich-Wintgen bound states in the continuum based on borophene metamaterials. Opt. Express.

[52] Chen Z.Y., Cheng S.B., Zhang H.F., Yi Z., Tang B., Chen J., Zhang J.G., Tang C.J. (2024). Ultra wideband absorption absorber based on Dirac semimetallic and graphene metamaterials. Phys. Lett. A.

[53] Li Y.M., Tan C.X., Hu J.Y., Bai W.D., Zhang R.L., Lin Q., Zhang Y., Wang L.L. (2022). Ultra-narrow band perfect absorbance induced by magnetic lattice resonances in dielectric dimer metamaterials. Results Phys..

[54] Ling Z.X., Zeng Y., Liu G.D., Wang L.L., Lin Q. (2022). Unified model for plasmon-induced transparency with direct and indirect coupling in borophene-integrated metamaterials. Opt. Express.

[55] Wang J.Q., Yang J.Y., Mei Y.W. (2025). Non-radiating anapole state in dielectric nanostructures and metamaterials. J. Phys. D Appl. Phys..

[56] Zeng Y., Ling Z.X., Liu G.D., Wang L.L., Lin Q. (2022). Tunable plasmonically induced transparency with giant group delay in gain-assisted graphene metamaterials. Opt. Express.

[57] Wang J., Yang H., Yi Z., Wang J., Cheng S., Li B., Wu P. (2025). High Absorption Broadband Ultra-Long Infrared Absorption Device Based on Nanoring–Nanowire Metasurface Structure. Photonics.

[58] Xiang T., Sun Z., Wang L.L., Lin Q., Liu G.D. (2024). Polarization independent perfect absorption of borophene metamaterials operating in the communication band. Phys. Scr..

[59] Cheng S.B., Li W.X., Zhang H.F., Akhtar M.N., Yi Z., Zeng Q.D., Ma C., Sun T.Y., Wu P.H., Ahmad S. (2024). High sensitivity five band tunable metamaterial absorption device based on block like Dirac semimetals. Opt. Commun..

[60] Gu X., Liu X., Yan X.F., Du W.J., Lin Q., Wang L.L., Liu G.D. (2023). Polaritonic coherent perfect absorption based on self-hybridization of a quasi-bound state in the continuum and exciton. Opt. Express.

[61] Li W., Yi Y., Yang H., Cheng S., Yang W.X., Zhang H., Yi Z., Yi Y., Li H. (2023). Active Tunable Terahertz Band-width Absorber Based on single layer Graphene. Commun. Theor. Phys..

[62] Li B.X., Liu M.L., Wen R.Q., Wei Y., Zeng L.L., Deng C.S. (2023). Dynamic control of Fano-like interference in the graphene periodic structure. J. Phys. D Appl. Phys..

[63] Yan X.F., Lin Q., Wang L.L., Liu G.D. (2023). Tunable strong plasmon–exciton coupling based modulator employing borophene and deep subwavelength perovskite grating. J. Phys. D Appl. Phys..

[64] Li Z., Song Q.J., Jia L.B., Yi Z., Cheng S.B., Wang J.Q., Li B.X. (2025). Actively tunable multi-frequency narrowband terahertz absorber using graphene metamaterials. Opt. Commun..

[65] Long T., Zhang L., Wang L.L., Lin Q. (2022). Tunable narrow transparency windows induced by the coupled quasi-guided modes in borophene plasmonic nanostructure. J. Phys. D Appl. Phys..

[66] Zeng T.Y., Liu G.D., Wang L.L., Lin Q. (2021). Light-matter interactions enhanced by quasi-bound states in the continuum in a graphene-dielectric metasurface. Opt. Express.

[67] Gu X., Liu G.D., Wang L.L., Lin Q. (2022). Robust Fano resonance induced by topologically protected interface modes interference at gigahertz. Appl. Phys. Express.

[68] Gao L., Zhu T., Deng M., Chiang K.S., Sun X., Dong X., Hou Y. (2012). Long-period fiber grating within d-shaped fiber using magnetic fluid for magnetic-field detection. IEEE Photonics J..

[69] Luo L., Pu S., Dong S., Tang J. (2015). Fiber-optic magnetic field sensor using magnetic fluid as the cladding. Sens. Actuators A.

[70] Thakur H.V., Nalawade S.M., Gupta S., Kitture R., Kale S.N. (2011). Photonic crystal fiber injected with Fe_3_O_4_ nanofluid for magnetic field detection. Appl. Phys. Lett..

[71] Zhao Y., Lv R.-Q., Ying Y., Wang Q. (2012). Hollow-core photonic crystal fiber Fabry-Perot sensor for magnetic field measurement based on magnetic fluid. Opt. Laser Technol..

[72] Bao L., Dong X., Zhang S., Shen C., Shum P. (2018). Magnetic field sensor based on magnetic fluid-infiltrated phase-shifted fiber Bragg grating. IEEE Sens. J..

[73] Huang H., Zhang Z., Yu Y., Zhou L., Tao Y., Li G., Yang J. (2020). A Highly Magnetic Field Sensitive Photonic Crystal Fiber Based on Surface Plasmon Resonance. Sensors.

